# Impact of patient characteristics and treatment procedures on hospitalization cost and length of stay in Japanese patients with influenza: A structural equation modelling approach

**DOI:** 10.1111/irv.12505

**Published:** 2017-10-27

**Authors:** Rosarin Sruamsiri, Sameh Ferchichi, Aurélien Jamotte, Mondher Toumi, Hiroshi Kubo, Jörg Mahlich

**Affiliations:** ^1^ Health Economics Janssen Pharmaceutical KK Tokyo Japan; ^2^ Center of Pharmaceutical Outcomes Research Naresuan University Phitsanulok Thailand; ^3^ Creativ‐Ceutical Paris France; ^4^ Public Health Department Aix‐Marseille University Marseille France; ^5^ Research & Development Department Janssen Pharmaceutical KK Tokyo Japan; ^6^ Düsseldorf Institute for Competition Economics (DICE) University of Düsseldorf Düsseldorf Germany

**Keywords:** economic burden, healthcare costs, hospitalizations, Influenza, Japan, structural equation modelling

## Abstract

**Objectives:**

Little is known about the economic burden of influenza‐related hospitalizations in Japan. This study sought to identify the factors that contribute to the total healthcare costs (THCs) associated with hospitalizations due to influenza in the Japanese population.

**Study design:**

A retrospective cross‐sectional database analysis study.

**Methods:**

A structural equation modelling approach was used to analyse a nationwide Japanese hospital claims data. This study included inpatients with at least 1 confirmed diagnosis of influenza and with a hospital stay of at least 2 days, who were admitted between April 2014 and March 2015.

**Results:**

A total of 5261 Japanese inpatients with a diagnosis of influenza were included in the final analysis. The elderly (≥65 years) and the young (≤15 years) comprised more than 85% of patients. The average length of stay (LOS) was 12.5 days, and the mean THC was 5402 US dollars (US$) per hospitalization. One additional hospital day increased the THC by 314 US$. Intensive care unit hospitalizations were linked to higher costs (+4957 US$) compared to regular hospitalizations. The biggest procedure‐related cost drivers, which were also impacted by LOS, were blood transfusions (+6477 US$), tube feedings (+3501 US$) and dialysis (+2992 US$).

**Conclusions:**

In Japan, the economic burden due to influenza‐related hospitalizations for both children and the elderly is considerable and is further impacted by associated comorbidities, diagnostic tests and procedures that prolong the LOS.

## 1 INTRODUCTION

Influenza is a contagious respiratory illness caused by a highly infectious viral pathogen. The illness ranges from mild to severe and can lead to numerous complications such as superimposed infections, exacerbation of cardiovascular conditions and asthma. Most of the fatal cases occur in the elderly over 65 years old^1^ and in high‐risk populations including children younger than 2 years old,^2^ pregnant women,^3^ healthcare workers^4^ and patients with associated comorbidities such as asthma, chronic lung disease, kidney disorders and blood disorders.^5^, ^6^

Annual epidemics of influenza result in approximately 250 000 to 500 000 deaths worldwide.^7^ Cases of influenza also have a substantial socioeconomic impact in terms of medical care, healthcare utilization (eg increase in consultations, hospitalizations and length of stay [LOS]) and work absenteeism.^8^ In Europe, influenza is responsible for approximately 10% of sickness‐related workplace absence.^9^ As influenza is an epidemic disease, it may disturb the healthcare services by acute overloading during the epidemic.

Elderly patients comprise the group with the highest burden of influenza‐related complications. Patients aged ≥85 years are 6 times more likely to be hospitalized and 16 times more likely to die compared with patients aged 65‐69 years.^10^, ^11^

The costs associated with influenza and its complications can be substantial. In the United States, a study based on the 2003 population estimated that the annual burden of influenza was 3.1 million hospital days and 31.4 million outpatient visits. From a societal perspective, the total economic burden of influenza (direct costs and indirect costs, including loss of earnings and loss of life) has been estimated to be 87.1 billion US$ annually, with direct costs accounting for more than 10 billion US$, of which 40% is spent on the treatment of patients older than 65 years of age.^12^

In the United States, the mean total cost of hospitalization for influenza‐related illness for children was 13 159 US$ (39 792 US$ for patients admitted to an intensive care unit (ICU) and 7030 US$ for patients cared for exclusively on the wards). High‐risk patients had a higher mean total cost than low‐risk patients (15 269 vs 9107 US$, respectively).^13^

In Japan, it is estimated that 5%‐10% of the population develops influenza annually, resulting in approximately 1000 to 2000 deaths from influenza alone and an additional 5000 deaths due to complications such as pneumonia.^14^ Approximately 20% to 25% of elderly Japanese patients with influenza develop pneumonia, 5% of whom die.^14^ From 1988 to 1991, 14.0% of all admissions to paediatric hospitals during the winter season in Japan were due to influenza viral infections, while respiratory syncytial virus accounted for 17.5% of admissions.^15^ Despite these statistics, there is limited information available about the extent of the disease burden due to influenza‐related illness in Japan. Therefore, the aim of this study was to identify factors that impact hospitalization costs for patients with influenza in Japan utilizing a Japanese administrative database.

## 2 METHODS

### 2.1 Patient selection

We utilized a commercially available hospital claims databank from Medical Data Vision Co., Ltd (MDV, Tokyo, Japan). This is an administrative database including approximately 4 400 000 patients, which represents approximately 3% of the total Japanese population.^16^ The MDV database has been used to investigate a wide range of conditions in Japan such as rheumatoid arthritis,^17^, ^18^ schizophrenia,^19^ infectious diseases,^20^ multiple sclerosis^21^ and hypertension.^22^ We considered the inpatient claims from patients who were admitted between 1 April 2014 and 31 March 2015 with at least 1 confirmed diagnosis of influenza [International Classification of Diseases 10th Revision (ICD‐10) codes: J10.1, J11.1 and J11.8] and a minimum hospital stay of 2 days (defined by at least 1 night was spent in the hospital).

### 2.2 Hospitalization cost calculation

Total healthcare costs (THCs) comprised all costs of healthcare services incurred during each hospitalization. These included basic management fees, examination, procedures and medication. Both Diagnosis Procedure Combination cost (DPC cost, which is a case‐mix reimbursement cost) and total actual heath care cost were calculated. All costs were converted from Japanese yen to US$ based on the average exchange rate during April 2014‐March 2015 (Financial Market Department, Bank of Japan; 1 US$ = 109.33 yen).^23^

### 2.3 Statistical analysis

**Figure 1:**
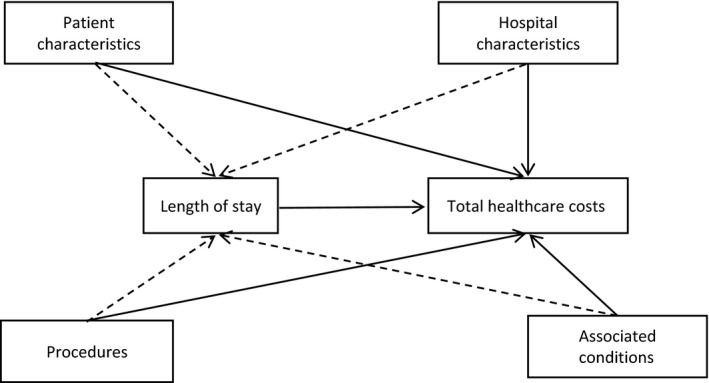
Path showing the relationship between each variable using structural equation modelling. Patient characteristics: age, gender, origin of patients and main diagnosis. Hospital characteristics: hospital type (regular, ER, ICU). Procedures: blood transfusion, cardiac catheterization, dialysis, mechanical ventilator, oxygen therapy, tube feeding, biochemical testing, bronchoscopy, chest X‐ray, echocardiography, CT scan, immunology test, sputum test and oxygen saturation test. Associated conditions: congestive heart failure, atrial fibrillation, acute respiratory failure, pneumonia, asthma, COPD, chronic renal failure, diabetes mellitus, disease involving the immune mechanism, Parkinson's disease, ischaemic heart disease and malignant neoplasm (cancer). Indirect effect: dashed line. Direct effect: continuous line. ER, emergency; ICU, intensive care unit; CT scan, computerized axial tomography scan; COPD, chronic obstructive pulmonary disease

Descriptive analyses were performed on baseline characteristics as well as resource use, LOS and THC. As LOS is usually an important driver of the total hospitalization costs,^24^, ^25^ we considered a structural equation modelling (SEM) approach to assess the relationship between the patients’ characteristics, procedures, LOS and hospitalization costs by considering LOS as an intermediate effect. Indeed, SEM is a flexible multivariate statistical framework that can be used to model complex relationships between variables.^26^ The SEM framework allows evaluating relationships among variables by combining the strengths of factor analysis and multiple regression in a single model that can be tested statistically.^27^ More specifically, in this study, a path analysis was conducted, which is a special case of the SEM framework that allows an exploration of the causal links (direct and indirect effects) between exogenous variables and 1 or more endogenous variables. In this framework, the total effects of a covariate on the main dependent variable can be decomposed into 2 categories of effects: (i) the indirect effects, consisting of the effect of the covariate on 1 or more intermediary endogenous variables, which in turn translates into an effect on the main variable; and (ii) the direct effect, which is the remaining effect of the covariate on the main variable while controlling for their indirect effects.^28^ In our case, the main endogenous variable of interest in the analysis was the total hospitalization cost expressed in Japanese yen, while we assumed that independent variables would have both a direct effect on total hospitalization costs and indirect effects through the LOS. Figure 1 depicts the underlying path diagram showing the relationship between each variable. We also conducted subgroup analyses of the children (≤15 years), the elderly (≥65 years) populations and the infants and toddlers (children ≤2 years old), 3 groups that are particularly susceptible to influenza complications and hospitalization. Statistical analyses were performed using stata 15.0.^29^

**Figure 2:**
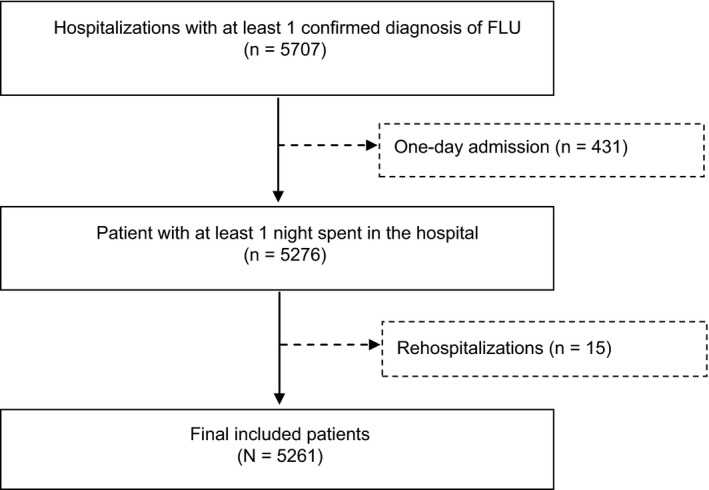
Patient selection

## 3 RESULTS

A total of 5261 Japanese inpatients with influenza were included in the final analysis. We excluded 15 rehospitalized admissions due to the limited number of patients (Figure 2).

Table 1 shows patient baseline characteristics for all patients and each subgroup. The elderly (≥65 years) and children (≤15 years) were 61.8% and 26.1% of the patients, respectively. Overall, the average length of hospital stay was 12.5 days, and the mean THC was 5402 US$. 4.5% of the patients were admitted to an ICU, and 4.7% of the patients died in the hospital. The most prominent comorbidities were diabetes (14.9%), congestive heart failure (13.1%) and pneumonia (13.1%). A computerized tomography (CT) scan was used as a diagnostic aid in 49.9% of patients, 44.0% of patients received oxygen therapy, approximately 5.5% of patients received a blood transfusion during their hospitalization, 5.6% received tube feeding and 4.1% required mechanical ventilation.

**Table 1:** Characteristics of included influenza patients

Characteristics N (%)	Total N (%)	Children (≤15 y) N (%)	Adults (16‐64 y) N (%)	Elderly (≥65 y and older) N (%)	Subgroup: Infants and toddlers (≤2 y) N (%)
Number of patients	5261	1375 (26)	637 (12)	3249 (62)	654 (12)
Demographics					
Gender					
Female	2559 (49)	567 (41)	303 (47)	1689 (52)	276 (42)
Age					
Mean ± SD (median [Q1; Q3])	57.5 ± 34.9 (75 [12; 85])	4.0 ± 3.8 (3 [1; 7])	45.6 ± 14.6 (49 [35; 59])	82.5 ± 8.0 (83 [77; 88])	0.8 ± 0.8 (1 [0; 2])
Hospitalization characteristics					
Influenza as diagnosis incurring most resources	1867 (35)	884 (64)	130 (20)	853 (26)	436 (67)
Influenza as primary medical diagnosis	2343 (44)	924 (67)	169 (26)	1250 (38)	445 (68)
Nature of hospitalization					
Regular	3033 (58)	1196 (87)	385 (61)	1452 (45)	566 (87)
Emergency	1990 (38)	161 (12)	205 (32)	1624 (50)	79 (12)
ICU	238 (4)	18 (1)	47 (7)	173 (5)	9 (1)
Origin of patient before hospitalization					
Hospitalized from home	4708 (89)	1355 (98)	612 (96)	2714 (84)	643 (98)
Transfer	91 (2)	10 (1)	8 (1)	73 (2)	6 (1)
Nursing home or welfare facilities	436 (8)	0 (0)	11 (2)	425 (13)	0 (0)
Missing	26 (1)	10 (1)	6 (1)	10 (1)	5 (1)
Destination/outcome after discharge					
Home	4208 (80)	1357 (99)	595 (93)	2256 (70)	647 (99)
Transfer	349 (7)	5 (0)	18 (3)	326 (10)	2 (0)
Long‐term care facilities	414 (8)	0 (0)	12 (2)	402 (12)	0 (0)
Death	248 (5)	1 (0)	8 (1)	239 (7)	1 (0)
Missing	42 (0)	12 (1)	4 (1)	26 (1)	4 (1)
Associated conditions					
Congestive heart failure	690 (13)	9 (1)	41 (6)	640 (80)	7 (1)
Atrial fibrillation	305 (6)	0 (0)	8 (1)	297 (9)	0 (0)
Acute respiratory failure	535 (10)	67 (5)	40 (6)	428 (13)	29 (4)
Acute renal failure	65 (1)	3 (0)	14 (2)	48 (1)	1 (0)
Pneumonia	689 (13)	114 (8)	43 (7)	532 (16)	59 (9)
Asthma	562 (11)	265 (19)	59 (9)	238 (7)	121 (10)
COPD	285 (5)	1 (0)	21 (3)	263 (8)	0 (0)
Chronic renal failure	196 (4)	1 (0)	26 (4)	169 (5)	1 (0)
Diabetes mellitus	785 (15)	0 (0)	97 (15)	688 (21)	0 (0)
Disease involving the immune mechanism	11 (0)	3 (0)	4 (1)	4 (0)	0 (0)
Parkinson's disease	82 (2)	2 (0)	3 (0)	77 (2)	0 (0)
Ischaemic heart disease	405 (8)	1 (0)	33 (5)	371 (11)	0 (0)
Malignant neoplasm (cancer)	503 (10)	7 (1)	78 (12)	418 (13)	1 (0)
Procedures (patients with at least 1 procedure charged)					
Surgery and interventions					
Blood transfusion	288 (6)	10 (1)	40 (6)	238 (7)	4 (1)
Cardiac catheterization	943 (18)	12 (1)	78 (12)	853 (26)	4 (1)
Dialysis	73 (1)	0 (0)	18 (3)	55 (2)	0 (0)
Mechanical ventilation	217 (4)	22 (2)	28 (4)	167 (5)	11 (2)
Oxygen therapy	2317 (44)	227 (16)	201 (31)	1888 (58)	107 (16)
Tube feeding	296 (6)	18 (1)	20 (3)	258 (8)	6 (1)
Other surgery procedures and anaesthesia	839 (16)	53 (4)	159 (25)	627 (19)	19 (3)
Tests/imaging					
Biochemical testing	5092 (97)	1277 (93)	604 (95)	3210 (99)	603 (92)
Bronchoscopy/pulmonary function test	142 (3)	9 (1)	28 (4)	105 (3)	2 (0)
Chest X‐ray	4598 (87)	919 (67)	540 (85)	3139 (97)	418 (91)
Colour Doppler ultrasound/echocardiography	1174 (22)	76 (6)	150 (24)	948 (29)	29 (4)
Computerized tomography scan	2597 (49)	235 (17)	288 (45)	2074 (64)	81 (12)
Immunology test	4690 (89)	996 (72)	575 (90)	3119 (96)	481 (73)
Oxygen saturation test	2551 (48)	384 (28)	223 (35)	1944 (60)	186 (28)
Sputum test	3046 (58)	687 (50)	313 (49)	2046 (63)	328 (58)
Length of stay					
LOS					
Mean ± SD (median [Q1; Q3])	12.5 ± 12.7 (8 [4; 17])	4.3 ± 5.4 (3 [2; 5])	11.3 ± 11.0 (7 [4; 14])	16.1 ± 13.5 (12 [6; 22])	4.2 ± 5.1 (3 [2; 5])
Total costs in USD					
1. Total costs (sum of costs of all procedures)					
Mean ± SD	5402 ± 5597	2619 ± 3500	5572 ± 6146	6546 ± 5793	2538 ± 3629
Median [Q1; Q3]	3409 [2036; 6638]	1937 [1570; 2546]	3491 [2120; 6687]	4669 [2793; 8224]	1935 [1607; 2441]
2. Total cost of DPC					
Mean ± SD	4582 ± 5075	2265 ± 3144	4770 ± 5675	5526 ± 5296	2205 ± 3198
Median [Q1; Q3]	2718 [1635; 5566]	1679 [1363; 2185]	2744 [1536; 5817]	3821 [2124; 6900]	1686 [1393; 2124]

DPC, Diagnosis Procedure Combination; SD, standard deviation; ICU, intensive care unit; COPD, chronic obstructive pulmonary disease; LOS, length of stay; ¥, Japanese yen; USD, US$.

Exchange rate: 1 USD = 109.33 Japanese yen.

The results of the SEM method are reported in Table 2.

**Table 2:** Direct, indirect and total effects of the factors on THC using a structure equation model

Variable	Direct effect (USD^a^)			Indirect effect (USD^a^)			Total effects (USD^a^)		
	→THC			→LOS→THC			→THC + (→LOS→THC)		
	Coeff.	95% CI		Coeff.	95% CI		Coeff.	95% CI	
LOS (day)	**314**	**297**	**330**				**314**	**297**	**330**
Gender (reference: male)									
Female	**−176**	**−307**	**−45**	**259**	**94**	**424**	82	−125	291
Age (reference: 16‐64 y)									
≤15 y	**457**	**196**	**719**	**−484**	**−753**	**−216**	−26	−429	375
16‐64 y	**Reference**			**Reference**			**Reference**		
65 y and older	**−473**	**−764**	**−182**	**854**	**580**	**1127**	**381**	**−33**	**795**
Hospitalization characteristics									
Influenza as primary medical diagnosis	**−415**	**−543**	**−287**	**−1579**	**−1767**	**−1390**	**−1994**	**−2195**	**−1793**
Nature of hospitalization									
Regular	**Reference**			**Reference**			**Reference**		
Emergency	**459**	**326**	**593**	−261	−462	−61	197	−33	429
ICU	**4769**	**3915**	**5623**	188	−384	762	**4957**	**3832**	**6083**
Patient origin									
From home	**Reference**			**Reference**			**Reference**		
Transfer	−330	−906	246	763	−120	1647	433	−423	1290
Nursing home or welfare facilities	**−685**	**−898**	**−472**	209	−151	569	**−476**	**−853**	**−98**
Associated conditions									
Congestive heart failure	−139	−446	167	218	−108	544	78	−347	505
Atrial fibrillation	−246	−651	157	28	−407	464	−218	−758	322
Acute respiratory failure	−64	−315	186	−314	−639	10	−379	−769	11
Acute renal failure	1001	−713	2716	−881	−1777	13	119	−1849	2088
Pneumonia	**−405**	**−597**	**−213**	−41	−306	223	−446	−748	−144
Asthma	−177	−406	52	97	−148	343	−79	−428	269
COPD	**−330**	**−590**	**−71**	288	−143	720	−42	−500	414
Chronic renal failure	**−1042**	**−1728**	**−357**	141	−495	779	**−900**	**−1782**	**−19**
Diabetes mellitus	141	−114	398	110	−161	383	252	−111	617
Disease involving the immune mechanism	1499	−2046	5046	−1379	−2709	−49	120	−4389	4630
Parkinson's disease	−128	−533	277	**1755**	**799**	**2710**	**1626**	**680**	**2573**
Ischaemic heart disease	**516**	**99**	**934**	334	−50	720	**851**	**339**	**1363**
Malignant neoplasm (cancer)	**464**	**124**	**804**	**997**	**597**	**1397**	**1462**	**904**	**2019**
Procedures (patients with at least 1 procedure charged)									
Surgery and interventions									
Blood transfusion	**3557**	**2846**	**4268**	**2919**	**2231**	**3608**	**6477**	**5379**	**7575**
Cardiac catheterization	24	−242	292	**1744**	**1388**	**2101**	**1769**	**1348**	**2191**
Dialysis	**2453**	**1109**	**3797**	539	−483	1561	**2992**	**1311**	**4673**
Mechanical ventilation	**2435**	**1618**	**3252**	**−718**	**−1355**	**−80**	**1717**	**678**	**2756**
Oxygen therapy	**301**	**83**	**519**	199	−68	468	**501**	**149**	**853**
Tube feeding	**881**	**240**	**1522**	**2619**	**2028**	**3210**	**3501**	**2639**	**4362**
Tests/imaging									
Biochemical testing	56	−178	291	−304	−619	9	−248	−656	160
Bronchoscopy/pulmonary function test	**2032**	**1045**	**3020**	**1449**	**717**	**2181**	**3482**	**2218**	**4746**
Chest X‐ray	**282**	**114**	**450**	109	−90	309	**391**	**117**	**666**
Colour Doppler ultrasound/echocardiography	**538**	**338**	**739**	**972**	**690**	**1254**	**1511**	**1174**	**1848**
Computerized tomography	19	−130	170	**588**	**392**	**785**	**608**	**362**	**853**
Immunology test	−64	−261	132	**420**	**218**	**622**	**355**	**31**	**680**
Oxygen saturation test	−67	−262	127	235	−2	474	168	−137	474
Sputum test	−90	−242	60	**529**	**358**	**700**	**438**	**207**	**669**

Statistical significance at *P*‐value < 0.05 in bold. Coeff., unstandardized coefficient; USD, US$; LOS, length of stay; THC, total healthcare cost; COPD, chronic obstructive pulmonary disease; ICU, intensive care unit.

a Exchange rate: 1 USD = 109.33 Japanese yen.

Results of the SEM analysis showed that hospitalizations where influenza was the primary diagnosis were 1994 US$ less costly than those with another medical diagnosis. One additional hospital day increased the THC by 314 US$. Not surprisingly, ICU stays were significantly more costly (+4957 US$) than regular stays. Among comorbidities, ischaemic heart disease, malignant neoplasm and Parkinson's disease significantly increased the THC by 851 US$, 1462 US$ and 1626 US$, respectively.

Overall, patients who were transferred from other hospitals incurred higher total costs; however, the opposite was found for toddlers under the age of 2. Patients who were referred from nursing home or welfare facilities are less costly than those who were hospitalized from home.

The majority of additional procedures were significantly associated with higher THC both directly and due to an increase in the LOS. Among surgeries and interventions, the largest cost drivers were blood transfusions (+6477 US$), tube feedings (+3501 US$) and dialysis (+2992 US$). Bronchoscopy and echocardiography were the imaging procedures that increased the THC most significantly (+3482 and +1511 US$, respectively). Overall, the effects on DPC costs compared with total costs were similar (Data S1).

Subgroup analyses of children (≤15 years) (Table 3), the elderly (≥65 years) (Table 4) and the infants and toddlers (≤2 years old) (Table 5) showed similar results, although the magnitude of the effect was higher in children for most of the surgeries and interventions.

**Table 3:** Direct, indirect and total effects of the factors on THC in children (≤15 y old) using a structure equation model

Variable	Direct effect (USD^a^)			Indirect effect (USD^a^)			Total effects (USD^a^)		
	→THC			→LOS→THC			→THC + (→LOS→THC)		
	Coeff.	95% CI		Coeff.	95% CI		Coeff.	95% CI	
LOS (day)	**549**	**485**	**612**				**549**	**485**	**612**
Gender (reference: male)									
Female	−44	−140	52	1	−240	243	−42	−292	207
Hospitalization characteristics									
Influenza as primary medical diagnosis	81	−37	199	**−980**	**−1295**	**−665**	**−899**	**−1208**	**−589**
Nature of hospitalization									
Regular	**Reference**			**Reference**			**Reference**		
Emergency	**336**	**230**	**443**	−73	−517	370	263	−165	692
ICU	**2688**	**979**	**4398**	140	−5917	6198	2829	−4505	10 164
Patient origin									
From home	**Reference**			**Reference**			**Reference**		
Transfer	−203	−581	173	−126	−731	478	−330	−791	130
Nursing home or welfare facilities	**Omitted**			**Omitted**			**Omitted**		
Procedures (patients with at least 1 procedure charged)									
Surgery and interventions									
Blood transfusion	**3865**	**37**	**7692**	**13 070**	**2433**	**23 707**	**16 935**	**3624**	**30 246**
Cardiac catheterization	764	−2491	4020	5278	−4391	14 948	6043	−5899	17 986
Mechanical ventilation	848	−320	2018	2003	−1088	5094	2851	−973	6677
Oxygen therapy	−104	−267	59	**860**	**373**	**1347**	**756**	**259**	**1254**
Tube feeding	914	−453	2282	−2856	−7346	1634	−1941	−7233	3350
Tests/imaging									
Biochemical testing	145	−37	329	−348	−718	22	−202	−552	147
Bronchoscopy/pulmonary function test	755	−1534	3046	−378	−2282	1526	377	−3249	4005
Chest X‐ray	−9	−137	117	**306**	**22**	**589**	**296**	**16**	**576**
Colour Doppler ultrasound/echocardiography	391	−116	899	**2068**	**770**	**3366**	**2460**	**998**	**3922**
Computerized tomography	**323**	**148**	**498**	61	−314	436	384	−14	784
Immunology test	45	−80	171	40	−291	371	85	−249	420
Oxygen saturation test	88	−25	202	−179	−379	20	−91	−302	120
Sputum test	1	−88	91	**319**	**49**	**588**	**320**	**52**	**588**

Statistical significance at *P*‐value < 0.05 in bold. Coeff., unstandardized coefficient; USD, US$; LOS, length of stay; THC, total healthcare cost.

a Exchange rate: 1 USD = 109.33 Japanese yen.

**Table 4:** Direct, indirect and total effects of the factors on THC in elderly patients (≥65 y old) using a structure equation model

Variable	Direct effect (USD^a^)			Indirect effect (USD^a^)			Total effects (USD^a^)		
	→THC			→LOS→THC			→THC + (→LOS→THC)		
	Coeff.	95% CI		Coeff.	95% CI		Coeff.	95% CI	
LOS (day)	**293**	**279**	**307**				**293**	**279**	**307**
Gender (reference: male)									
Female	−201	−385	−17	**471**	**238**	**703**	269	−28	567
Hospitalization characteristics									
Influenza as primary medical diagnosis	**−526**	**−690**	**−361**	**−1920**	**−2160**	**−1680**	**−2446**	**−2716**	**−2176**
Nature of hospitalization									
Regular	**Reference**			**Reference**			**Reference**		
Emergency	**443**	**283**	**603**	−265	−556	673	177	−109	464
ICU	**4943**	**3966**	**5920**	58	−556	673	**5002**	**3732**	**6272**
Patient origin									
From home	**Reference**			**Reference**			**Reference**		
Transfer	−332	−929	265	815	−179	1810	482	−515	1481
Nursing home or welfare facilities	**−662**	**−875**	**−448**	124	−221	470	**−537**	**−922**	**−152**
Associated conditions									
Congestive heart failure	−216	−514	81	156	−171	484	−60	−493	373
Atrial fibrillation	−198	−589	192	48	−368	465	−150	−687	387
Acute respiratory failure	−33	−289	222	−345	−712	22	−379	−799	41
Acute renal failure	1093	−958	3145	−715	−1679	248	377	−1967	2723
Pneumonia	**−406**	**−645**	**−168**	−41	−358	274	**−448**	**−824**	**−72**
Asthma	−48	−518	422	26	−423	475	−21	−699	656
COPD	**−316**	**−601**	**−30**	287	−159	733	**−28**	**−542**	**484**
Chronic renal failure	**−1009**	**−1516**	**−502**	58	−595	713	**−950**	**−1693**	**−207**
Diabetes mellitus	182	−82	448	73	−207	355	256	−132	645
Disease involving the immune mechanism	5115	−3837	14 067	−874	−3519	1770	4240	−7000	15 481
Parkinson's disease	11	−386	409	**1574**	**643**	**2505**	**1586**	**585**	**2586**
Ischaemic heart disease	271	−132	675	**336**	**−53**	**726**	**608**	**82**	**1134**
Malignant neoplasm (cancer)	259	−83	603	**723**	**333**	**1112**	**982**	**453**	**1512**
Procedures (patients with at least 1 procedure charged)									
Surgery and interventions									
Blood transfusion	**3354**	**2608**	**4099**	**2583**	**1936**	**3231**	**5938**	**4882**	**6993**
Cardiac catheterization	70	−196	338	**1516**	**1171**	**1862**	**1587**	**1167**	**2008**
Dialysis	**2123**	**1232**	**3014**	659	−524	1842	**2782**	**1288**	**4276**
Mechanical ventilation	**2519**	**1590**	**3448**	−630	−1316	55	**1888**	**741**	**3035**
Oxygen therapy	241	−53	536	150	−204	505	392	−86	870
Tube feeding	**1014**	**366**	**1662**	**2563**	**1956**	**3169**	**3578**	**2691**	**4464**
Tests/imaging									
Biochemical testing	114	−357	586	−460	−1313	392	−345	−1318	627
Bronchoscopy/pulmonary function test	**2222**	**1123**	**3322**	**1636**	**766**	**2506**	**3859**	**2407**	**5311**
Chest X‐ray	248	−124	621	**733**	**249**	**1218**	**982**	**374**	**1590**
Colour Doppler ultrasound/echocardiography	**553**	**338**	**768**	**753**	**455**	**1051**	**1306**	**947**	**1665**
Computerized tomography	−20	−213	172	**533**	**292**	**774**	**513**	**204**	**821**
Immunology test	37	−324	399	**1166**	**711**	**1620**	**1203**	**619**	**1788**
Oxygen saturation test	−51	−334	231	258	−83	600	207	−246	660
Sputum test	−212	−431	6	**474**	**241**	**708**	262	−58	582

Statistical significance at *P*‐value < 0.05 in bold. Coeff., unstandardized coefficient; USD, US$; LOS, length of stay; THC, total healthcare cost; COPD, chronic obstructive pulmonary disease.

a Exchange rate: 1 USD = 109.33 Japanese yen.

**Table 5:** Direct, indirect and total effects of the factors on THC in infants and toddlers (≤2 y old) using a structure equation model

Variable	Direct effect (USD^a^)			Indirect effect (USD^a^)			Total effects (USD^a^)		
	→THC			→LOS→THC			→THC + (→LOS→THC)		
	Coeff.	95% CI		Coeff.	95% CI		Coeff.	95% CI	
LOS (day)	**547**	**489**	**604**				**547**	**489**	**604**
Gender (reference: male)									
Female	34	−48	117	54	−136	245	88	−95	272
Hospitalization characteristics									
Influenza as primary medical diagnosis	8	−114	131	**−675**	**−940**	**−410**	−667	−899	−434
Nature of hospitalization									
Regular	**Reference**			**Reference**			**Reference**		
Emergency	**393**	**286**	**501**	−124	−395	147	269	1	537
ICU	**2229**	**525**	**3922**	777	−1659	3213	3006	−605	6618
Patient origin									
From home	**Reference**			**Reference**			**Reference**		
Transfer	−424	−969	121	31	−621	685	−392	−994	209
Nursing home or welfare facilities	**Omitted**			**Omitted**			**Omitted**		
Procedures (patients with at least 1 procedure charged)									
Surgery and interventions									
Blood transfusion	**5714**	**−80**	**11 509**	**21 181**	**8702**	**33 659**	**26 895**	**10 267**	**43 523**
Cardiac catheterization	2940	−1132	7012	**15 770**	**7533**	**24 007**	18 710	7647	29 773
Mechanical ventilation	641	−594	1877	1178	−131	2488	**1819**	**163**	**3476**
Oxygen therapy	−119	−321	81	**1226**	**636**	**1817**	**1106**	**523**	**1689**
Tube feeding	262	−3626	4152	−12 563	−25 823	696	−12 300	−29 057	4456
Tests/Imaging									
Biochemical testing	**207**	**30**	**384**	−205	−572	161	−1	−304	307
Bronchoscopy/pulmonary function test	4844	−3196	12 886	3309	−1341	7960	8154	−3524	19 832
Chest X‐ray	50	−36	138	**351**	**127**	**574**	**402**	**194**	**609**
Colour Doppler ultrasound/echocardiography	605	134	1076	**2343**	**964**	**3722**	**2949**	**1428**	**4470**
Computerized tomography	114	−114	343	**−540**	**−887**	**−194**	−426	−854	1
Immunology test	7	−83	98	99	−93	293	107	−261	276
Oxygen saturation test	86	−63	236	−208	−458	41	−121	−391	147
Sputum test	−80	−172	11	−26	−256	203	−106	−322	119

Statistical significance at *P*‐value < 0.05 in bold. Coeff., unstandardized coefficient; USD, US$; LOS, length of stay; THC, total healthcare cost.

a Exchange rate: 1 USD = 109.33 Japanese yen.

## 4 DISCUSSION

Using an administrative database of hospitalized Japanese patients with influenza, we found that influenza‐related hospitalizations mostly consisted of elderly and young patients, confirming that these 2 age groups are at high risk of influenza complications.

### 4.1 Impact of comorbidities on THC

It is not surprising that healthcare costs significantly increase when influenza strikes in association with other medical disorders. Our data revealed that Parkinson's disease had the highest impact on cost although it represented only 1.6% of the population, followed by cancer and ischaemic heart disease, which were 9.6% and 7.7% of the cases, respectively. The increased healthcare cost is most likely a reflection of the high incidence of influenza‐related complications that occur with these comorbidities.

Despite the low incidence of neurologic disorders associated with influenza viral infection, patients have a high risk of developing complications.^30^, ^31^ In addition, Parkinson's disease has been reported to be a clinical manifestation of influenza,^32^ and parkinsonian‐like symptoms such as tremors have also been described in severe influenza cases.^33^ Of note, influenza A is one of several viruses that have been implicated in the pathogenesis of Parkinson's disease.^34^, ^35^ Although a causal link has been difficult to establish in humans,^31^, ^34^, ^36^ a reduction in neuropsychiatric reactions in influenza patients treated with the antiviral oseltamivir suggests that the influenza virus may play a role in the pathogenesis of certain neurologic symptoms.^37^

Cancer patients are susceptible to infections such as influenza because of either treatment‐associated immunosuppression or the type of malignancy.^38^ These patients are also at high risk of developing influenza‐related complications. A German study that included 203 patients who had influenza along with haematologic and solid tumours reported a high rate of pneumonia and bacterial or fungal superinfections.^39^ Influenza also appears to have a detrimental impact on the outcome of cancer treatment by delaying the initiation of anticancer therapy.^40^

Chronic heart disease is one of the highest predictors of influenza‐related hospitalizations and complications.^41^ Epidemiologic studies have long reported an association between influenza epidemics and cardiovascular disease (CVD). For instance, acute myocardial infarctions (AMI) have their highest incidence in the winter months and are often preceded by an upper respiratory tract infection.^42^ In addition to the influenza‐related increase in hospitalizations for CVD, influenza is also linked to both increases in AMI^43^ and AMI‐related deaths.^44^, ^45^ Influenza infection has also been associated with damage to the heart muscle leading to cardiomyopathy and myocarditis.^46^ Taken together, these observations are consistent with our findings that patients with heart disease comprised a significant share of influenza‐related hospitalizations, and heart disease was an important driver of the increase in THC.

### 4.2 Role of patient origin

It was found that patients who were referred from nursing home or welfare facilities incurred less cost than those who were hospitalized from home. One possible interpretation of this interesting finding is that institutions such as nursing homes or welfare facilities do monitor their clients well and send them to the hospital even in case of a mild form of the disease. Elderly who live at home, on the other hand, might miss the right timing to seek medical advice.

### 4.3 Impact of procedures and ER and ICU admissions on THC

The most significant cost drivers among procedures were blood transfusions and tube feedings, which increased the THC by 380 000 Japanese yen (approximately 3450 US$).

Our findings of a high cost burden associated with ICU or ER admissions when compared with routine hospitalizations are consistent with other reports. In European countries, for instance, the daily cost of ICU admissions ranged from €1168 to €2025 (1240 to 2150 US$),^47^ while in the United States the estimated additional cost was 2190 US$ per day.^48^ These statistics underscore the importance of avoiding ICU or ER admissions whenever possible.

### 4.4 A role of vaccinations and antiviral treatment

The potential policy implication of our findings is that vaccination programmes should be promoted to avoid influenza‐related hospitalizations. From 1977 to 1987, there was already a vaccination programme for Japanese schoolchildren that achieved between 50% and 85% annual coverage in children aged 3‐15 years. It was shown that this vaccination programme was associated with a decrease in the overall number of influenza‐related excess deaths and that excess deaths increased once the programme was discontinued.^49^ Furthermore, because the vaccination of schoolchildren can reduce influenza‐related morbidity and mortality among non‐immunized contacts as well as the elderly, it was estimated that the vaccination programme could also save 1000 elderly lives per year.^50^

For those patients still requiring hospitalization, medical treatment may be an option to reduce hospital LOS and healthcare costs. A recent study in the United States that included 1 557 437 cases of influenza from 4 influenza seasons found an overall 11% reduction in the risk of complications in oseltamivir‐treated patients (an 81% reduction in those treated <2 days after the diagnosis).^31^ Antiviral treatment also decreased the risk of hospitalizations and emergency room visits by 29% and 24%, respectively. A recent cost‐effectiveness analysis in the Japanese healthcare context, for instance, demonstrated that treatment with oseltamivir was highly cost‐effective with an incremental cost‐effectiveness ratio (ICER) of 398 571 Japanese yen (3645 US$) per quality‐adjusted life year from a health insurance perspective. With the inclusion of productivity costs, the ICER for oseltamivir turned negative, meaning that medical treatment with oseltamivir was both cost‐saving and more effective.^51^

### 4.5 Limitations

There are several limitations to our study. First, this analysis is based on a 1‐year database. Thus, we could not capture the potential changes due to prescribing behaviour changes and the change of treatment guideline over time. Second, due to the limitations of the database, potentially useful information that might explain costs was lacking. For instance, we could not retrieve hospital ID numbers, which could have been used to identify heterogeneity between hospitals as well as patient characteristics such as region, social and professional status and clinical severity of their disease. Nevertheless, our analysis examined all available patient characteristics (such as age, gender and relevant comorbidities) that could be retrieved from the database. Third, bias may have resulted from the current DPC system that allows hospitals to choose the diagnosis that is incurring the most medical resource utilization as the main diagnosis. In general, patients with comorbidities will receive a higher reimbursement if hospitals choose comorbidities as the primary diagnosis. Finally, a major limitation of this study is that influenza‐related hospitalizations can be difficult to identify because influenza is not always detected as the primary cause of the hospitalization, especially in severe cases.^10^ As a result, this study may underestimate the true burden of influenza as well as the cost of influenza‐related hospitalizations because of the coding incentive. Only hospitalizations with influenza diagnosis, which are less costly, were included.^10^

## ETHICAL APPROVAL

The study was in line with the guidelines provided by Johnson & Johnson and was approved by the Janssen Approval Committee.

## CONFLICT OF INTEREST

JM, KH and RS are affiliated with Janssen Pharmaceutical KK, a pharmaceutical company. SF and AJ are employees of Creativ‐Ceutical, which received funding from Janssen Pharmaceutical KK to perform the study.
