# Supplementary material for: Impact of patient characteristics and treatment procedures on hospitalization cost and length of stay in Japanese patients with influenza: A structural equation modelling approach
Source: Influenza Other Respir Viruses. 2017 Oct 27;11(6):543–55. doi: 10.1111/irv.12505 (PMC5705683; doi:10.1111/irv.12505)
Supplement: Supplementary file 1 [file IRV-11-543-s001.docx]

**Data S1** Direct, indirect and total effects of the factors on **DPC costs** using a structure equation model

| **Variable** | | **Direct effect (USD*)** | | | **Indirect effect (USD*)** | | | | **Total effects (USD*)** | | |
| --- | --- | --- | --- | --- | --- | --- | --- | --- | --- | --- | --- |
|  |  | **🡪THC** | | | **🡪LOS🡪THC** | | | | **🡪THC + (🡪LOS🡪THC)** | | |
|  |  | **Coff.** | **95% CI** | | **Coff.** | | **95% CI** | | **Coff.** | **95% CI** | |
| **LOS (day)** | | **289** | **274** | **304** |  | | | | **289** | **274** | **304** |
| Gender (Reference Male) | |  |  |  |  |  | |  |  |  |  |
|  | Female | -94 | -219 | 31 | **239** | **86** | | **392** | 145 | -51 | 341 |
| Age (Reference 16 - 64 years) | |  |  |  |  |  | |  |  |  |  |
|  | ≤ 15 years | **496** | **239** | **752** | **-448** | **-695** | | **-200** | **47** | **-332** | **428** |
|  | 16 - 64 years | **Reference** | | | **Reference** | | | | **Reference** | | |
|  | 65 years and older | **-432** | **-720** | **-144** | **789** | **535** | | **1,042** | 356 | -33 | 747 |
| **Hospitalisation characteristics** | |  |  |  |  |  | |  |  |  |  |
| influenza as primary medical diagnosis | | **-498** | **-612** | **-383** | **-1,459** | **-1,632** | | **-1,285** | **-1,957** | **-2,145** | **-1,769** |
| Nature of hospitalisation | |  |  |  |  |  | |  |  |  |  |
|  | Regular | **Reference** | | | **Reference** | | | | **Reference** | | |
|  | Emergency | **377** | **250** | **503** | **-241** | **-427** | | **-56** | 135 | -80 | 350 |
|  | ICU | **4,714** | **3,871** | **5,557** | 174 | -355 | | 703 | **4,888** | **3,804** | **5,972** |
| **Patient origin** | |  |  |  |  |  | |  |  |  |  |
|  | From home | **Reference** | | | **Reference** | | | | **Reference** | | |
|  | Transfer | -134 | -686 | 417 | 705 | -112 | | 1,522 | 570 | -315 | 1,457 |
|  | Other facility | **-609** | **-813** | **-405** | 193 | -139 | | 526 | **-416** | **-772** | **-60** |
| **Associated conditions** | |  |  |  |  |  | |  |  |  |  |
|  | Congestive heart failure | -10 | -315 | 295 | 201 | -99 | | 503 | 191 | -220 | 603 |
|  | Atrial fibrillation | -309 | -630 | 11 | 26 | -376 | | 429 | -282 | -762 | 196 |
|  | Acute respiratory failure | -176 | -391 | 38 | -290 | -591 | | 9 | -467 | -817 | -117 |
|  | Acute renal failure | 305 | -866 | 1,478 | -814 | -1,642 | | 13 | -509 | -2,065 | 1,047 |
|  | Pneumonia | **-468** | **-621** | **-316** | -38 | -282 | | 206 | -506 | -770 | -243 |
|  | Asthma | -156 | -389 | 76 | 90 | -137 | | 317 | -65 | -411 | 280 |
|  | COPD | **-376** | **-628** | **-123** | 266 | -132 | | 665 | -109 | -529 | 310 |
|  | Chronic renal failure | **-791** | **-1,402** | **-181** | 131 | -458 | | 720 | -660 | -1,452 | 130 |
|  | Diabetes mellitus | -46 | -280 | 186 | 102 | -148 | | 353 | 55 | -274 | 385 |
|  | Disease involving the immune mechanism | 284 | -1,403 | 1,972 | -1,274 | -2,504 | | -44 | -989 | -3,644 | 1,664 |
|  | Parkinson disease | -105 | -525 | 313 | **1,621** | **739** | | **2,503** | **1,515** | **633** | **2,397** |
|  | Ischaemic heart disease | **721** | **304** | **1,138** | 309 | -47 | | 666 | **1,031** | **518** | **1,543** |
|  | Malignant neoplasm (cancer) | **410** | **91** | **729** | **921** | **554** | | **1,289** | **1,332** | **825** | **1,839** |
| **Procedures (patients with at least one procedure charged)** | |  |  |  |  |  | |  |  |  |  |
| Surgery and interventions | |  |  |  |  |  | |  |  |  |  |
|  | Blood transfusion | **3,053** | **2,406** | **3,699** | **2,697** | **2,067** | | **3,328** | **5,751** | **4,747** | **6,754** |
|  | Cardiac catheterisation | -200 | -472 | 71 | **1,612** | **1,286** | | **1,938** | **1,411** | **1,010** | **1,811** |
|  | Dialysis | **2,249** | **1,041** | **3,457** | 498 | -446 | | 1,442 | **2,747** | **1,159** | **4,334** |
|  | Mechanical ventilation | **2,077** | **1,372** | **2,783** | -663 | -1,252 | | -74 | **1,313** | **463** | **2,364** |
|  | Oxygen therapy | **290** | **80** | **500** | **184** | **-63** | | **433** | **475** | **144** | **805** |
|  | Tube feeding | **727** | **145** | **1,309** | **2,420** | **1,871** | | **2,969** | **3,147** | **2,321** | **3,973** |
| Tests/Imaging | |  |  |  |  |  | |  |  |  |  |
|  | Biochemical testing | -73 | -297 | 149 | -281 | -571 | | 8 | -355 | -744 | 33 |
|  | Bronchoscopy / Pulmonary  function test | **1,892** | **917** | **2,867** | **1,339** | **663** | | **2,014** | **3,231** | **2,012** | **4,449** |
|  | Chest X-Ray | **215** | **60** | **369** | **100** | **-83** | | **285** | **316** | **60** | **572** |
|  | Color Doppler Ultrasound/ echocardiography | **268** | **70** | **465** | **898** | **639** | | **1,157** | **1,166** | **850** | **1,483** |
|  | Computerized tomography | **-253** | **-400** | **-105** | **543** | **362** | | **725** | **290** | **59** | **521** |
|  | Immunology test | -137 | -334 | 60 | **388** | **202** | | **574** | 251 | -63 | 566 |
|  | Oxygen saturation test | -95 | -282 | 91 | 217 | -2 | | 438 | 122 | -164 | 409 |
|  | Sputum test | **-165** | **-307** | **-22** | **489** | **331** | | **646** | **324** | **108** | **539** |

Coff.: Unstandardized coefficient, USD: US$ LOS: Length of Stay, THC: Total health care cost.,COPD: chronic obstructive pulmonary disease

*Exchange rate: 1 USD = 109.33 Japanese Yen
